# Priorities for future research on reducing and stopping psychiatric medicines using a James Lind Alliance priority setting partnership: The PROTECT study protocol

**DOI:** 10.12688/hrbopenres.13649.1

**Published:** 2022-11-10

**Authors:** Miriam Boland, Agnes Higgins, Claire Beecher, Pat Bracken, Wendy Burn, Anne Cody, Adele Framer, Toto Anne Gronlund, Mark Horowitz, Christy Huff, Sandra Jayacodi, Dolores Keating, David Kessler, Asa Konradsson Geuken, Nicole Lamberson, Luke Montagu, Brian Osborne, Ruth Smith, Cathal Cadogan

**Affiliations:** 1 School of Pharmacy and Pharmaceutical Sciences, Trinity College Dublin, Dublin, Ireland; 2 School of Nursing and Midwifery, Trinity College Dublin, Dublin, Ireland; 3 Evidence Synthesis Ireland and Cochrane Ireland, University of Galway, Galway, Ireland; 4 School of Nursing and Midwifery, University of Galway, Galway, Ireland; 5 Independent Consultant Psychiatrist, West Cork, Ireland; 6 Past President of, Royal College of Psychiatrists, England, UK; 7 Consultant Psychiatrist, Leeds and York Partnership NHS Foundation Trust, England, UK; 8 Health Research Board, Dublin, Ireland; 9 SurvivingAntidepressants.org, California, USA; 10 James Lind Alliance, United Kingdom, UK; 11 Research and Development Department, Goodmayes Hospital, North East London NHS Foundation Trust, London, UK; 12 Benzodiazepine Information Coalition, Utah, USA; 13 Lived Experience Public Contributor, London, UK; 14 St John of God Hospital, Dublin, Ireland; 15 Centre for Academic Mental Health, Bristol Medical School, University of Bristol, England, UK; 16 Section of Neuropharmacology and Addiction Research, Department of Pharmaceutical Biosciences, Uppsala University, Sweden, Sweden; 17 European Federation of Associations of Families of People with Mental Illness, Belgium, Belgium; 18 International Institute for Psychiatric Drug Withdrawal, United Kingdom, UK; 19 Inner Compass Initiative's The Withdrawal Project, United States, USA; 20 Council for Evidence-based Psychiatry, United Kingdom, UK; 21 Irish College of General Practitioners, Dublin, Ireland; 22 Public Partner, England, UK

**Keywords:** Antidepressants, benzodiazepines, antipsychotics, psychotropic drugs, withdrawal, tapering, discontinuation, Patient and Public Involvement, Priority Setting Partnership, James Lind Alliance

## Abstract

**Background**: There is a growing number of service users looking to discontinue use of psychiatric medicines. Tapering is the recommended approach for reducing and/or discontinuing the use of psychiatric medicines. This involves gradually reducing the dose over time to minimise the potential for withdrawal symptoms. However, many uncertainties exist regarding the process of reducing and stopping psychiatric medicines. This study will use a James Lind Alliance Priority Setting Partnership to determine the Top 10 unanswered questions and uncertainties about reducing and stopping psychiatric medicines.

**Methods** **: **The Priority Setting Partnership will be conducted using the James Lind Alliance methodology. It will involve seven stages: (i) creating an international Steering Group of representatives from key stakeholder groups that will include people with lived experience of taking and/or stopping psychiatric medicines, family members, carers/supporters and healthcare professionals, and identifying potential partners to support key activities (e.g. dissemination); (ii) gathering uncertainties about reducing and stopping psychiatric medicines from key stakeholders using an online survey; (iii) data processing and summarising the survey responses; (iv) checking the summary questions against existing evidence and verifying uncertainties; (v) shortlisting the questions using a second online survey; (vi) determining the Top 10 research questions through an online prioritisation workshop; (vii) disseminating results.

**Conclusions** **: **This study will use a Priority Setting Partnership to generate a Top 10 list of research questions and uncertainties about reducing and stopping psychiatric medicines. This list will help to guide future research and deliver responsive and strategic allocation of research resources, with a view to ultimately improving the future health and well-being of individuals who are taking psychiatric medicines.

## Introduction

The total global consumption of psychiatric medicines (e.g., antidepressants, antipsychotics, benzodiazepine receptor agonists) has increased by 4% annually over an 11-year period spanning 2008–2019, with the greatest increase observed in antidepressant use ^1^. It is estimated that 7.8 million people in England received at least one prescription for an antidepressant in 2019/20, an increase of 13.9% from 2015/16 ( NHS, 2020). Similar figures have been reported in the United States whereby an estimated 13.2% of the adult population reported having recently taken antidepressants between 2015–2018 ( CDC, 2020).

Despite the widespread use of psychiatric medicines, the mechanism of action for drugs such as antidepressants is not fully understood. Previous theories suggesting that these drugs address an underlying chemical imbalance have been found to lack supporting evidence in some conditions ^2, 3^. The growing use of psychiatric medicines has attracted much attention and discussion. For example, the safety and efficacy of psychiatric medicines is increasingly being debated, particularly in terms of the extent of any clinically significant improvements in symptoms when compared to placebo, the potential for adverse effects and withdrawal symptoms, and the duration of treatment ^4– 6^. However, studies of individuals who have taken psychiatric medicines, such as antidepressants and antipsychotics, have highlighted a diversity of experiences ranging from positive to negative ^7– 11^. For example, one study that asked individuals taking antidepressants an open-ended question about their experience of taking antidepressants found that 54% (n=943) of respondents reported positive experiences, describing the medication as a ‘life-saver’ ^9^. This same study also found that 16% (n=279) of respondents had predominately negative experiences, whereby they found antidepressant treatment ineffective and experienced unpleasant side-effects ^9^. It has been postulated that polarised discourse as to whether psychiatric medicines work can be unhelpful and even confusing for those experiencing mental health challenges and that the importance of initiating conversations about the meaning of medicines in an individual’s life should not be overlooked ^12^.

There is a growing cohort of individuals looking to reduce and/or discontinue the long term use of psychiatric medicines ^13^. A recent study involving 269 people with schizophrenia and other psychotic disorders found that 31% of participants taking antipsychotics would like to try to stop medicines with professional help and 45% would like the opportunity to reduce the medicines ^14^. There are many reported motivations for discontinuing psychiatric medicines ^14– 18^. In some cases, these medicines may have failed to manage the person’s distressing experience and symptoms, while others may find the adverse effects intolerable. Some individuals will have recovered on medication and wish to see if they can stop it without relapsing. Common adverse effects of psychiatric medicines include weight gain, sexual dysfunction and akathisia (feeling of restlessness) ^19^. Some adverse effects are associated with specific classes of drugs. For example, tardive dyskinesia (involuntary movements) is associated with antipsychotics, in particular first generation antipsychotics ^20^.

One of the main barriers to successful discontinuation of psychiatric medicines such as antidepressants, antipsychotics and benzodiazepine receptor agonists is withdrawal symptoms. Withdrawal symptoms include anxiety, lethargy, tremors, mania, and delirium ^21– 24^. Withdrawal symptoms are not synonymous with the terms relapse or addiction ^21^. Withdrawal symptoms can be explained by the neurophysiologic readjustments that take place in the absence of these drugs ^25, 26^. A recent systematic review found that more than 50% of people experience withdrawal effects when they discontinue antidepressants ^23^. Withdrawal symptoms can be debilitating and protracted. Depending on their frequency and severity, these symptoms can form part of a withdrawal syndrome which can be acute or persistent in nature ^27– 30^. Withdrawal symptoms are one of the main contributing factors to increases in the long-term use of psychiatric medicines ^29, 31^.

Despite the growing number of individuals looking to discontinue psychiatric medicines, there is an absence of high-quality evidence underpinning the process of withdrawal ^14, 23^. Tapering is the approach that is typically employed by individuals attempting psychiatric discontinuation. Tapering involves a gradual dose reduction of the medicines over a prolonged period ^21, 32^. A conservative 10% dose reduction per month of the most recent dose is a common approach with some successes reported ^21, 22^. In April 2022, the National Institute for Health and Care Excellence (NICE) published guidelines which recommend tapering as the preferred method of discontinuation that involves *“a slow, stepwise rate of reduction proportionate to the existing dose”* ^33^. To date, there is no standard approach on how best to taper, which thereby creates many uncertainties regarding the tapering process ^34^. The lack of consistent guidance has also been seen as a barrier to discontinuation, with particular relevance to the prescriber ^31^. In the absence of comprehensive guidelines and effective interventions and supports, individuals are increasingly relying on online resources, such as discussion fora and Facebook groups, for guidance and support to deal with existing uncertainties relating to the tapering of psychiatric medicines ^21, 35– 37^.

The James Lind Alliance (JLA) is a UK-based non-profit initiative that has developed a Priority Setting Partnership (PSP) process to identify and prioritise unanswered questions i.e., ‘evidence uncertainties’ in specific conditions or areas of healthcare. This involves a working partnership between key stakeholder groups (people with lived experience of taking and/or stopping psychiatric medicines, family members, carers/supporters and healthcare professionals) ^38^. The study described in this protocol aims to determine the Top 10 unanswered questions and uncertainties reducing and stopping psychiatric medicines in collaboration with key stakeholders.

## Methods

**Figure 1.**
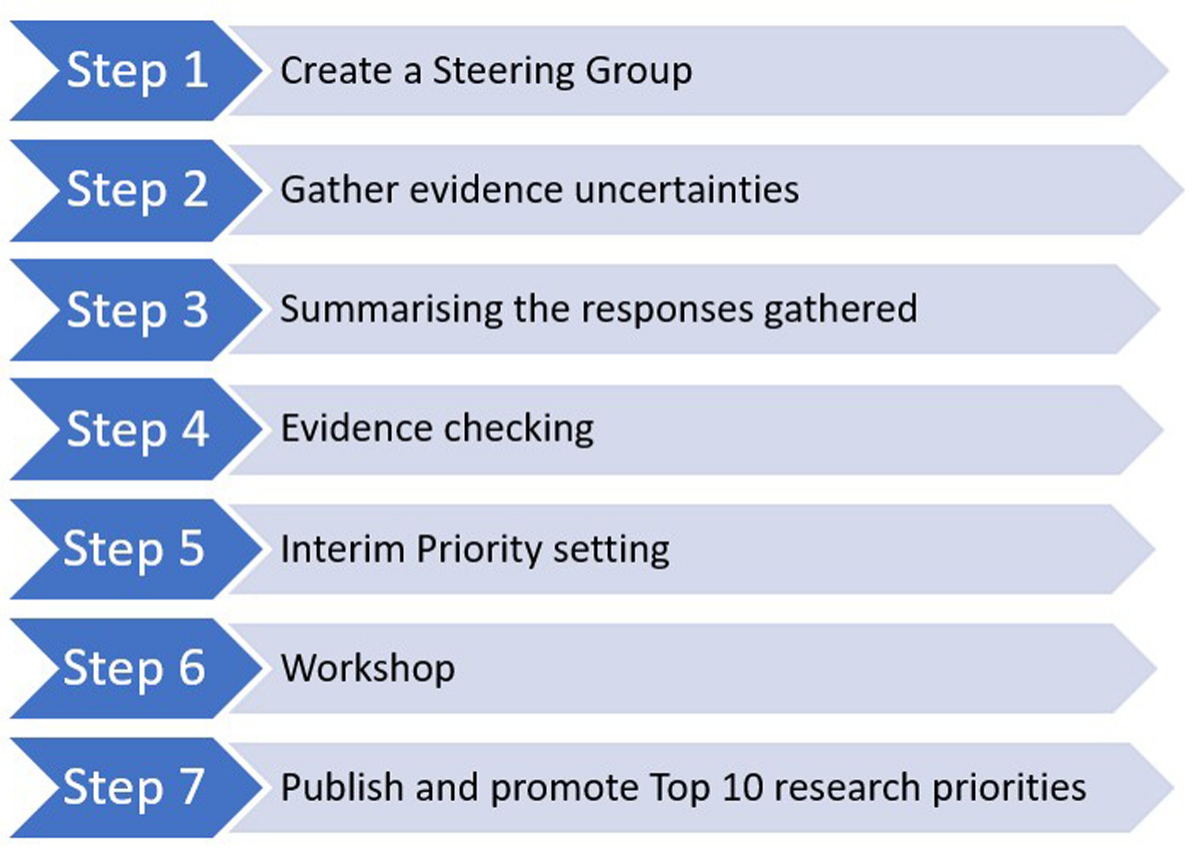
Overview of the Priority Setting Partnership (PSP) process.

This study will follow the JLA methodology ^38^. As illustrated in Figure 1 and outlined in more detail below, the PSP will comprise seven key steps. The first steps will involve establishing an international Steering Group to oversee the study and identifying potential partners to publicise the project. An anonymous online survey will then be conducted to gather key stakeholders’ uncertainties about reducing and stopping psychiatric medicines. Survey responses will be summarised, reviewed, and checked against existing evidence. Unanswered questions will be prioritised by key stakeholder groups in a second online survey. The most highly prioritised questions (approximately 25) will then be discussed as part of the final prioritisation workshop involving the Steering Group and additional collaborators representing the key stakeholder groups, and the Top 10 research priorities for reducing and stopping psychiatric medicines will be agreed. The results of the study will be disseminated through the JLA website and other networks as part of a detailed dissemination plan.

### Ethical approval

Ethical approval was granted by the Faculty of Health Sciences Research Ethics Committee, Trinity College Dublin on 3 ^rd^ August 2022 (reference number: 220509).

### Step 1: Create a Steering Group

An international Steering Group has been established to oversee and guide the PSP in accordance with JLA guidance ^38^. The Steering Group comprises representatives from key stakeholder groups (i.e., people with lived experience of taking and/or stopping psychiatric medicines, family members, carers/supporters, and healthcare professionals) from different countries. All Steering Group members are collaborators on the project (as opposed to research participants) and have co-authored this protocol. Their consent to being involved in the project is implicit in their voluntary attendance at Steering Group meetings. The main role of the Steering Group will involve overseeing and guiding the PSP process, which will include discussing and agreeing the PSP’s strategic orientation, in particular the scope of the PSP, i.e., the patient population of interest and the breadth of the topic ^38^. The Steering Group meetings will be held online on a regular basis to maintain transparency and promote engagement and momentum. These meetings will be chaired by an independent JLA advisor.

Potential Steering Group members were identified through an iterative approach whereby the researchers identified several key stakeholders to approach, through their network contacts, convened preliminary meetings with them to explain the aims of the project, answer any queries and invite recommendations for other Steering Group members based on their own networks and/or knowledge of other individuals who would be able to make a meaningful contribution to the project. This helped to ensure that the selection process was not solely informed by the researchers. At the first Steering Group meeting, a gap analysis of representation was conducted, and efforts made to purposely fill those gaps. No individuals affiliated with pharmaceutical industry were included in the Steering Group as per JLA rules ^38^.

Potential partners affiliated with key stakeholder groups (described above) will be identified and invited to support the PSP. The role of partners will be to disseminate information about the project to their members and affiliates, including dissemination of the online surveys (Steps 3&4) to potential respondents. Steering Group members will help to identify partners through their networks. Potential partners will be contacted by email, provided with summary information about the project and asked if they would be willing to support PSP related activities. The number of partners will be discussed and agreed by the Steering Group. No partners affiliated with pharmaceutical industry will be included in the study as per JLA rules ^38^.

### Step 2: Gather evidence uncertainties (initial online survey)

An anonymous online survey will be distributed to gather uncertainties about reducing and stopping psychiatric medicines from key stakeholders (people with lived experience of taking and/or stopping psychiatric medicines, family members, carers/supporters and healthcare professionals). The target population of survey respondents will be diverse and mirror the groups represented by individuals on the Steering Group. The psychiatric medicines of direct interest to this study will be antidepressants, antipsychotics, benzodiazepine receptor agonists, gabapentinoids, and mood stabilisers. The use of psychiatric medicines for both physical and mental health conditions will be considered within the scope of this PSP.

The survey will be designed using Qualtrics® survey software tool. Survey respondents will be asked to give their consent at the start of the survey by ticking a box to confirm that they are ≥18 years old, represent one of the key stakeholder groups described above and that they agree to complete the anonymous survey voluntarily. In the event that a respondent indicates that they do not consent to participating, they will be brought to the end of the survey. The survey will be piloted by the Steering Group members, followed by a small cohort of individuals representing the key stakeholder groups to ensure that the survey’s content is understandable by the wider population. Pilot responses will not be included in the analysis. The survey will consist of an open-ended question in line with the established JLA methods ^38^. This question will ask respondents to share their questions/uncertainties about reducing and stopping psychiatric medicines. Any questions related to the decision-making process of reducing and stopping psychiatric medicines, the methods used, the dose reductions and the duration, and the withdrawal symptoms, will be considered to be within the scope. Meanwhile, questions not related to psychiatric medicines will be considered as “out of scope”. The scope will be agreed and finalised by the Steering Group prior to the survey’s dissemination. Survey respondents will also be asked to provide some demographic information such as gender, age, country of residence, and representative group (i.e., which stakeholder group best represents the respondent). This is to ensure that a diverse cohort of respondents is obtained.

There are no formal target sample sizes for responses to a PSP survey. The survey will be open for approximately 4–6 weeks depending on the number of responses received. The number of survey responses being returned by stakeholder groups will be monitored on a weekly basis and if any stakeholder group participation reaches <10% of the total responders, efforts will be made to target the promotion of the survey towards the under-represented groups. The survey will be disseminated and promoted using a multi-strand approach. This will primarily involve social media (e.g., Twitter). Steering Group members and partners will also be asked to disseminate the survey through their own networks and websites.

### Step 3: Summarise the responses gathered

The responses from the initial online survey (Step 2) will consist of uncertainties and unanswered questions about reducing and stopping psychiatric medicines. Analysis of quantitative survey data will be conducted using SPSS version 16 and analysis of free-text response data will be conducted using qualitative content analysis ^39^. NVivo QSR 10 software will be used to aid free-text data management.

The questions/uncertainties will be categorised and refined by the research team (MB, CC, AH) into summary questions. Any questions classed as “out of scope” will be excluded from this list and kept separately. Similar or duplicate submissions will be combined where appropriate. The Steering Group will have oversight of the data analysis process to ensure that the raw survey data are being interpreted appropriately and transparently and that summary questions are clear and addressable by research, as well as understandable by all.

### Step 4: Evidence checking

The remaining “in scope” questions will be checked against existing sources of information, in particular systematic reviews, evidence-based guidelines, and prospective trial registers, to see to what extent these questions have, or have not, been addressed by previous or ongoing research. Questions that are not adequately addressed by previous research will be collated and recorded on a standard JLA template by the research team. These questions will be included in the second online survey for prioritisation by participants (Step 5).

### Step 5: Interim priority setting (second survey)

A second online survey will be created using Qualtrics ^®^ survey software tool, whereby the same groups of key stakeholders as per Step 3 will be asked to prioritise and shortlist the newly developed questions. Individuals can respond to the survey irrespective of whether they completed the previous survey. Participants will be asked to prioritise the questions whereby they will select ten questions that they feel are the most important and rank them in order of importance, using a scale of 1–10. The most highly prioritised questions (approximately 25) will be taken forward to the prioritisation meeting. The priorities of different categories of respondents will be listed separately and compared. If the long list is unduly long, the Steering Group will decide which questions are taken forward to the prioritisation meeting.

### Step 6: Workshop (final prioritisation)

A prioritisation workshop will be held virtually to prioritise through consensus the most highly ranked questions about reducing and stopping psychiatric medicines based on the second online survey. Steering Group members will be invited to attend the workshop and suggest additional colleagues/peers who would make a useful contribution to the meeting. The number of participants will be limited to 30, with representation across each of the stakeholder groups. The meeting will follow the standard approach described in the JLA handbook ^38^. In advance of the meeting, attendees will be provided with the short-listed questions from Step 5 to allow time to familiarise themselves with the questions. The meeting will use virtual breakout rooms to support small group discussions in which attendees will share their views and complete a number of ranking exercises. Whole group discussions will be used to reach a consensus and agree on a Top 10 list of research questions. The meeting will be facilitated by the JLA staff to ensure fairness, transparency, and accountability.

### Step 7: Publish and promote Top 10 research priorities

The results of the study will be published on the JLA website and will be reported to research funding and agenda setting organisations such as the Health Research Board (Ireland) and the Wellcome Trust (United Kingdom), as well as other relevant national and international organisations (e.g., mental health charities).

A comprehensive dissemination plan will be developed in consultation with the Steering Group. This plan will identify the target audiences and how to reach them and agree how Steering Group members and partners can engage with dissemination activities. A summary report and lay summary will be made available to all partner organisations involved and the public, a manuscript will be submitted for publication in a peer reviewed open access journal, and a pre-print will be uploaded to an open access repository for immediate access.

### Study status

At the time of submission, only Step 1 (create the Steering Group) has been completed. It is anticipated that Step 2 will be completed by the end of 2022 and that the remaining Steps will be completed by end of 2023.

## Discussion

Research prioritisation is an important means of minimising research waste and ensuring that research resources are targeted towards answering the most important questions ^40^. Following the James Lind Alliance process, this study will use a Priority Setting Partnership to generate a Top 10 list of research questions and uncertainties about reducing and stopping psychiatric medicines with active input from key stakeholders (people with lived experience of taking and/or stopping psychiatric medicines, family members, carers/supporters, and healthcare professionals). This work is particularly important given that use of psychiatric medicines is increasing, driven to a large extent by increases in the number of people taking the medicines for extended periods. Despite the growing number of people looking to discontinue their psychiatric medicines, there is a sparsity of high-quality evidence on the process of withdrawal ^31^. While tapering is the most recommended approach, the lack of robust evidence to inform guidance has created many uncertainties about the tapering process and which interventions would best support individuals who wish to discontinue psychiatric medicines ^31, 34^. This lack of evidence also creates challenges for healthcare practitioners who wish to provide safe evidence-based care. The Top 10 research questions and uncertainties identified by the PROTECT study will help to guide future research and deliver responsive and strategic allocation of research resources, with a view to ultimately improving the future health and well-being of people who are taking psychiatric medicines.

### Dissemination of findings

The completed review will be submitted for publication in a peer-reviewed journal.

## Data Availability

No underlying data are associated with this article. Open Science Framework: Priorities for future research on reducing and stopping psychiatric medicines using a James Lind Alliance priority setting partnership: The PROTECT study protocol. https://doi.org/10.17605/OSF.IO/RC9PY ^41^ This project contains the questions for the first survey. Data are available under the terms of the Creative Commons Zero "No rights reserved" data waiver (CC0 1.0 Public domain dedication).
