# Supplementary material for: Priorities for future research on reducing and stopping psychiatric medicines using a James Lind Alliance priority setting partnership: The PROTECT study protocol
Source: HRB Open Res. 2022 Nov 10;5:72. [Version 1] doi: 10.12688/hrbopenres.13649.1 (PMC10450262; doi:10.12688/hrbopenres.13649.1)
Supplement: Peer review history

# Peer review: Reviewer response for version 1

**DOI:** 10.21956/hrbopenres.14926.r35761

**Published:** 2023-08-24

**Authors:** Marie Crowe

**Affiliations:** 1 University of Otago, NZ, New Zealand

Thank you for the opportunity to review this study protocol. It is an important and long-overdue study that will have important impacts across primary care and tertiary services. Many of those with lived experiences while reluctant to take psychiatric medications, are also unsure how to stop taking them and often receive unhelpful advice from the medical profession. I cannot imagine other areas of medicine would continue to prescribe medications with such limited effect and with such a raft of side-effects that many are not told about on commencement of prescription.

 This is a well-designed study that can provide important data for those with lived experience. Given the lack of credible information available about how these medications work, I think it would be interesting to include questions how both clinicians and those with lived experience think they work and where they got their information.

 Good luck with this well-designed and important study.

Is the study design appropriate for the research question?

Yes

Is the rationale for, and objectives of, the study clearly described?

Yes

Are sufficient details of the methods provided to allow replication by others?

Yes

Are the datasets clearly presented in a useable and accessible format?

Yes

Reviewer Expertise:

Mood disorders

I confirm that I have read this submission and believe that I have an appropriate level of expertise to confirm that it is of an acceptable scientific standard.

# Peer review: Reviewer response for version 1

**DOI:** 10.21956/hrbopenres.14926.r34415

**Published:** 2023-07-14

**Authors:** Donna Goodridge

**Affiliations:** 1 College of Medicine, University of Saskatchewan, Saskatoon, SK, Canada

Using a priority-setting approach for the discontinuation of psychiatric medications is very appropriate to ascertain the views of stakeholders, particularly in light of the many controversies in this area.

 The introduction provides excellent justification for the rationale for this project with appropriate citations. The international composition of the Steering Group is commendable.

 I would be interested in further details of the recruitment and marketing strategy for the initial online survey beyond social media. In our own experience, we found that significant effort was required to recruit participants using a range of strategies. 

 Plans for analysis of the initial survey data and evidence-checking are appropriate. The interim priority setting and workshop plans follow the accepted JLA protocol.

Is the study design appropriate for the research question?

Yes

Is the rationale for, and objectives of, the study clearly described?

Yes

Are sufficient details of the methods provided to allow replication by others?

Yes

Are the datasets clearly presented in a useable and accessible format?

Not applicable

Reviewer Expertise:

Post-COVID Condition, Obstructive Sleep Apnea

I confirm that I have read this submission and believe that I have an appropriate level of expertise to confirm that it is of an acceptable scientific standard.
